# Temporal Control of Seed Development in Dicots: Molecular Bases, Ecological Impact and Possible Evolutionary Ramifications

**DOI:** 10.3390/ijms22179252

**Published:** 2021-08-26

**Authors:** Yury V. Malovichko, Anton E. Shikov, Anton A. Nizhnikov, Kirill S. Antonets

**Affiliations:** 1 Laboratory for Proteomics of Supra-Organismal Systems, All-Russia Research Institute for Agricultural Microbiology (ARRIAM), 196608 St. Petersburg, Russia; yu.malovichko@arriam.ru (Y.V.M.); a.shikov@arriam.ru (A.E.S.); a.nizhnikov@arriam.ru (A.A.N.); 2 Faculty of Biology, St. Petersburg State University, 199034 St. Petersburg, Russia

**Keywords:** plants, dicots, seed, development, timing, embryogenesis, heterochrony

## Abstract

In flowering plants, seeds serve as organs of both propagation and dispersal. The developing seed passes through several consecutive stages, following a conserved general outline. The overall time needed for a seed to develop, however, may vary both within and between plant species, and these temporal developmental properties remain poorly understood. In the present paper, we summarize the existing data for seed development alterations in dicot plants. For genetic mutations, the reported cases were grouped in respect of the key processes distorted in the mutant specimens. Similar phenotypes arising from the environmental influence, either biotic or abiotic, were also considered. Based on these data, we suggest several general trends of timing alterations and how respective mechanisms might add to the ecological plasticity of the families considered. We also propose that the developmental timing alterations may be perceived as an evolutionary substrate for heterochronic events. Given the current lack of plausible models describing timing control in plant seeds, the presented suggestions might provide certain insights for future studies in this field.

## 1. Introduction

**Figure 1:**
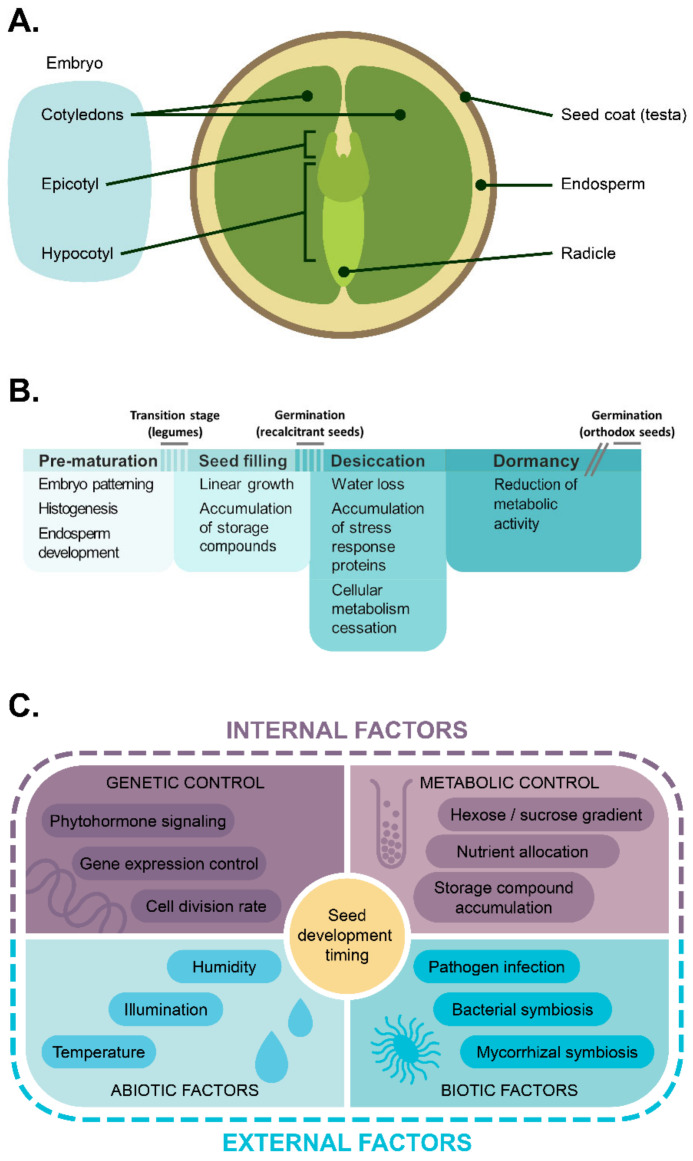
An overview of legume seed anatomy and maturation mechanisms. (**A**) Simplified anatomy of legume seed. (**B**) Timeline of seed development. The overlaps between stage bars reflect the coincidence of several processes intrinsic for different stages in some plants, e.g., family Brassicaceae. The break in the bar denoting the dormancy stage refers to the (potentially) unlimited duration of dormancy in desiccated orthodox seeds. (**C**) Key regulators of seed development and dormancy reviewed in this paper.

Among the land plants, spermatophytes, or seed plants, represent the most diverse and thriving lineage. This evolutionary success could, to a certain extent, be attributed to the formation of seeds serving as agents of both propagation and dissemination through securing the embryo’s endurance and providing it with nutrients at the onset of life. In flowering plants, the typical seed anatomy comprises three crucial elements: a seed coat, an endosperm, and an embryo, the former one arising from maternal tissues solely and the latter two being products of fertilization [1] (Figure 1A). By default, the flowering plant seeds start their development with the double fertilization event. The following events form a timeline divided into several stages (Figure 1B). At the first stage of seed ontogeny, the future embryo pattern and symmetry are established [2], accompanied by the development of the endosperm [3]. At the following stage, the seed synthesizes the necessary nutrient storage pool and acquires the potency to germinate [4,5]. In the so-called recalcitrant seeds, this stage indicates the end of the pre-germinative phase of seed development; such seeds are observed in plants dwelling in humid environments [6,7,8]. Most flowering plants, however, produce orthodox seeds, which further undergo a desiccation stage, acquiring the necessary endurance to external stimuli and then proceeding to the dormant state [5]. This generalized plan is subject to variations throughout flowering plants [9,10], which confounds both the generalization of developmental outline and devising common terminology. First, the early stage of seed development referring to embryo patterning is often called pre-storage or pre-maturation as opposed to bona fide maturation, since the onset of storage acquisition is thought to come primarily after the patterning process is over [11]. In accordance with this, a specific interim period, denoted as the transition stage, is distinguished in some plants, such as legumes (family Fabaceae), during which hormonal and metabolic control circuits are switched from maternal to filial ones within the seed [12]. However, embryo patterning and maturation may also overlap, as in the case of *Arabidopsis thaliana* (hereafter *Arabidopsis*), in which both embryo cell division and embryo morphogenesis overlap with the maturation-associated events [4]. Moreover, the term ‘maturation’ may refer either to seed filling or to both seed filling and seed desiccation, depending on whether these processes resolve sequentially, as in legumes, or overlap, as in members of the Brassicaceae family (see citation [5] and references therein). The notation we stick to in this review is reflected in Figure 1B.

Despite a plethora of mechanisms affecting seed development in flowering plants, they can be, for heuristic purposes, reduced to a simple scheme involving several key components (Figure 1C). Two principal ramifications of this scheme regard the internal or external origin of developmental stimuli. The internal factors involved in seed development mostly comprise the phytohormonal signaling [11,13,14,15,16], as well as genetic [17,18,19,20,21] and epigenetic control [22,23,24,25,26]. Apart from these mechanisms, small compounds, such as sugars [27,28,29], and lipid synthesis intermediates [30,31] may exert both metabolic and signaling functions. The external stimuli, in their turn, are provided by both abiotic, such as temperature, humidity, and light [32,33,34,35], and biotic [36] factors.

While stage succession is primarily conserved across flowering plants domain, the duration of the particular stages and overall seed development varies both within and between species. For instance, in crop plants, the traits related to the time needed for seed maturing, such as days to maturity (DTM) and days from flowering to maturity (DFTM), are crucial as they define the timing of crop harvesting. Subsequently, the genome-wide association studies (GWAS) of crop species frequently include searching for the quantitative trait loci (QTL) of DTM and DFTM heredity. Only in legumes, analyses of loci controlling either DTM, DTFM, or both, features were carried out for *Glycine max* (soybean) [37,38,39], *Phaseolus vulgaris* (common bean) [40], *Vigna angularis* (adzuki bean) [41], and *Pisum sativum* (garden pea) [42]. In plant biotechnology, the task of seed developmental cycle compression was addressed in a series of works [43,44]. However, neither of these approaches addresses the problem of developmental timing directly, although they provide certain clues on the underlying molecular mechanisms. The resulting dearth of data on seed developmental timing control suggests that this problem remains in its infancy and needs further clarification and conceptualization.

In this review, we summarize the existing data on the mechanisms of seed developmental timing control in dicots. Most of the experimental results in this account come from the model plants belonging to families Brassicaceae and Fabaceae, thus restraining the scope of the review. To mitigate these restrictions, the most general processes of seed development were selected for scrutiny to pinpoint the commonalities between the dicot families. The scrutinized phenotypes refer to two major sources of variability, namely, genetic mutations and adaptive responses to environmental stimuli. For the sake of convenience, the reviewed cases of genetically conditioned alterations were grouped depending on the mechanisms they violate, be that cellular division during early embryogenesis, endoreduplication and growth of embryo cells, maturation onset and progression, endosperm development, mitochondria and plastids’ maintenance, or storage compound synthesis (for the list of mutations, see Table S1). Based on that, we suggest the common trends of temporal alterations in seed development and how they may point out the mechanisms behind developmental timing control.

## 2. Cell Proliferation during Embryogenesis

**Figure 2:**
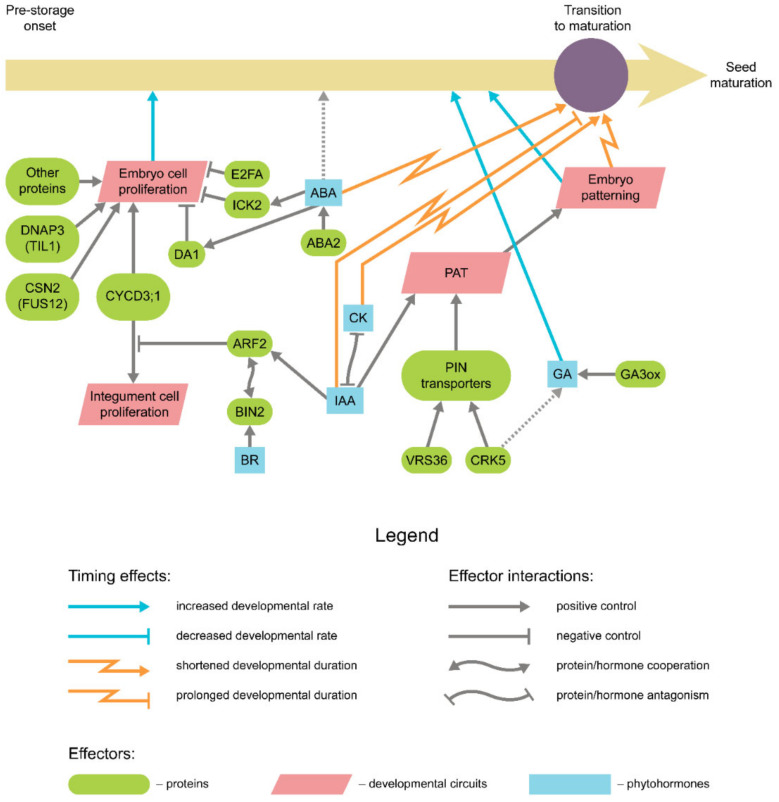
Main genetic and hormonal factors affecting pre-storage progression in dicots. For arrow shape and color meaning, see the figure legend. Abbreviations stand for: *IAA*—auxin, *CK*—cytokinin, *ABA*—abscisic acid, *GA*—gibberellin. The promoting effect of ABA on cell proliferation was proposed in references [53,54]. For CRK5-mediated coupling of IAA and GA signaling, see reference [55]. PAT—polar auxin transport.

Apparently, cell proliferation occurs in seeds predominantly at the pre-storage phase. At the onset of embryogenesis, the so-called proembryo stage, cell divisions are tightly linked to the establishment of the embryo polarity and patterning. Hormonal control of these primary divisions, especially by auxin, was adequately studied (reviewed in references [45,46,47]) (Figure 2); however, the impact of respective mutations usually involves a drastic decrease in the embryo viability up to the point of the seed abortion. The following rounds of cell division are, apparently, less restricted in their number and duration and thus may serve as the source of temporal plasticity. Since in eudicots the initial number of cotyledon cells contributes mostly to the final seed size [48], the dimensional seed characteristics and developmental timing are often tightly interconnected to the point of correlation [49], the latter having been observed in *Vicia faba* (broad bean) [50], *Medicago truncatula* (barrel medic) [51], and *P. sativum* [52].

During transition to maturation, the cells cease proliferation in favor of endoreduplication. This switch involves a recurrent progression through the G1/S checkpoint with no subsequent chromatid segregation, nucleus (karyokinesis), and cell (cytokinesis) division. The complex machinery of transition from the regular cell cycle to the endoreduplication has been described elsewhere [56,57,58]. Here we would like to emphasize that the necessity of passing the G1/S transition and S phase indicates at least partial similarity of mechanisms between these two programs. In their turn, the mutations affecting these mechanisms would alter the timing of both pre-storage and early maturation stages. The mutations of the *TIL1* gene in *Arabidopsis* encoding DNA polymerase ε were found to prolongate the duration of the S phase of the cell cycle [59]. The mutant *til1* embryos completed their development with a lower cell number, albeit at larger cell and embryo size. Apart from that, the overall seed development timing is also delayed in *til1* mutants regarding the chronological age but not the developmental age [59,60]. Among the mechanisms involved in G1/S transition licensing, D3 subfamily cyclins and COP9 signalosome were shown to affect development speed if mutated. The triple D3-type cyclin loss-of-function mutants of *Arabidopsis* demonstrate slower development at the pre-storage phase, while the overexpression led to an increased size at the reduced seed viability [61]. In somatic tissues, overexpression of *CYCD3* genes promotes cell division and represses endoreduplication [62], while the loss-of-function mutations vice versa lead to elevated levels of endoreduplication and restrained cell proliferation [63]. The *fus12* mutants impaired in the CSN2 subunit of the COP9 signalosome also display slower embryo growth as a result of G1/S transition delay [64,65,66].

Positive control of cell proliferation during embryogenesis relies on several phytohormonal circuits. Auxin is usually assumed to promote cell divisions in proliferating tissues [67]. The enhanced auxin production was recorded in highly heterozygous hybrids of *V. faba*, resulting in prolonged cell divisions and delayed transition phase [68]. An impairment of auxin gradient observed in *Arabidopsis vps36* vesicular trafficking mutants led to a similar delay in development, although no seed size alteration was reported [69]. In addition, the auxin is also known to repress the cell cycle development through the expression of *AUXIN RESPONSE FACTOR 2* (*ARF2*), whose product represses the cell divisions in the ovule tissues [70]. Notably, *arf2* mutation in *Arabidopsis* leads to prolonged expression of *CYCD3;1* genes in vegetative tissues [70]. This might be the cause of phenotype observed in *Arabidopsis arf2* seeds, which are larger yet develop at a slower pace as compared to wild-type seeds, although the spurious nature of *ARF2* expression in filial tissues suggests that this effect is mostly attributed to an enlarged seed cavity. Furthermore, the mode of action for ARF2 involves interaction with BRASSINOSTEROID INSENSITIVE 2 (BIN2) kinase [71], indicating possible synergy of these two hormones in the negative control of cell proliferation.

Compared to auxin, the roles of cytokinin and gibberellin in eudicot embryo development are less characterized. In *P. sativum*, the *LH* locus mutations encoding *ent*-kaurene oxidase, one of the key enzymes of the GA synthesis pathway, lead to the embryo growth rate debilitation and frequent seed abortion [72,73]. Being apparently unrelated to nutrient distribution, this effect is likely to be connected to the cell division rate [73]. Recently, GA and auxin signaling pathways have been shown to be interconnected in *Arabidopsis* embryo development via the activity of CRK5 kinase [55]. Mutations in *AtCRK5* led to decreased synthesis of active gibberellin forms and distortion of auxin gradient accompanied by the growth retardation and diminishing of linear embryo size. Cytokinin was shown to accumulate during embryo development in *P. sativum*, predominantly in the form of *cis*-isomers, and promote embryo growth [74]. Moreover, the elevated levels of isopentenyl riboside were found to accumulate during the embryo cell proliferation in accessions of *M. truncatula* with the prolonged pre-storage duration [51].

By the end of embryogenesis, high ABA levels trigger an arrest of the cell divisions in the embryo, indicating the onset of the transition phase [4,75]. The proposed mechanisms for this include repression of *CYCD3* and *CYC2A* genes via activating the *ICK* expression [76]. Alternatively, ABA can activate the DA1 ubiquitin receptor, which also negatively affects cell proliferation [77]. DA1-dependent degradation pathway includes DA2 protein that being impaired was reported to prolong the embryo proliferation phase in *Arabidopsis* [78]. Prior to the transition stage, however, some constitutive levels of ABA are required to maintain a proper cell division rate [4]. In *Arabidopsis*, ABA-deficient *aba2* mutants had been reported to produce smaller embryos due to the arrest of both cell division and cell expansion [53], although later research did not corroborate this notion [54]. Notably, large-seeded accessions of *M. truncatula* were also demonstrated to accumulate ABA with no penalty to the embryo proliferation [51]. It was demonstrated that the pre-storage stage duration, in this case, is sustained by the elevated auxin concentrations, suggesting that the ABA/auxin ratio may form a specific circuit of pre-storage duration control [51].

## 3. Endoreduplication and Cell Expansion

Starting from the transition stage, embryo growth is achieved predominantly by the cell expansion and endoreduplication in cotyledon cells [52,79]. Endomitoses usually start prior to the storage accumulation and coincide with both the residual cell division phase and cell expansion phase onset [80]. The reports on their hormonal control in cotyledon cells appear scanted (see reference [81], Section 3.1.7.2.3, for a thorough review). Cytokinins are known to bolster the onset of endoreduplication in the somatic tissues [82,83]. In turn, auxin promotes regular cell divisions and represses endocycles through TIR1-AUX/IAA-ARF signal transduction system in the root meristem of *Arabidopsis* [82]. A similar effect of auxin on the switch to endomitoses was confirmed for *M. truncatula* seeds [84]. In the latter case, however, the external application of auxins was found not only to postpone but also to prolong endoreduplication in the *M. truncatula* cotyledon seeds. This indicates that to a first approximation, a prolonged or enhanced auxin supplement may increase the seed development time and, collaterally, the seed size. In spite of this, in legumes the transition phase-associated auxin peak is claimed to coincide with the endoreduplication onset [35,85]. Whether these discrepancies reflect the lack of correlation between the programs governing the embryo and endosperm development or imply the differences between elevated auxin concentration per se and decreased cytokinin/auxin ratio requires further elucidation.

The evidence for cell growth and expansion affecting temporal seed progression is comparably rare. One example is the *EXS* (*EMS1*) gene of *Arabidopsis* encoding a receptor-like kinase with unknown functions, mutation of which leads to delayed seed development and reduced cell size without altering cell number [86]. A similar effect was observed for the mutation in the marneral synthase locus *MRN1* of *Arabidopsis,* with effects presumably caused by increased membrane permeability [87]. Although cell expansion is expected to affect the seed size rather than developmental timing, further studies may reveal a tighter connection between these features.

## 4. Genetic Control of Seed Maturation

**Figure 3:**
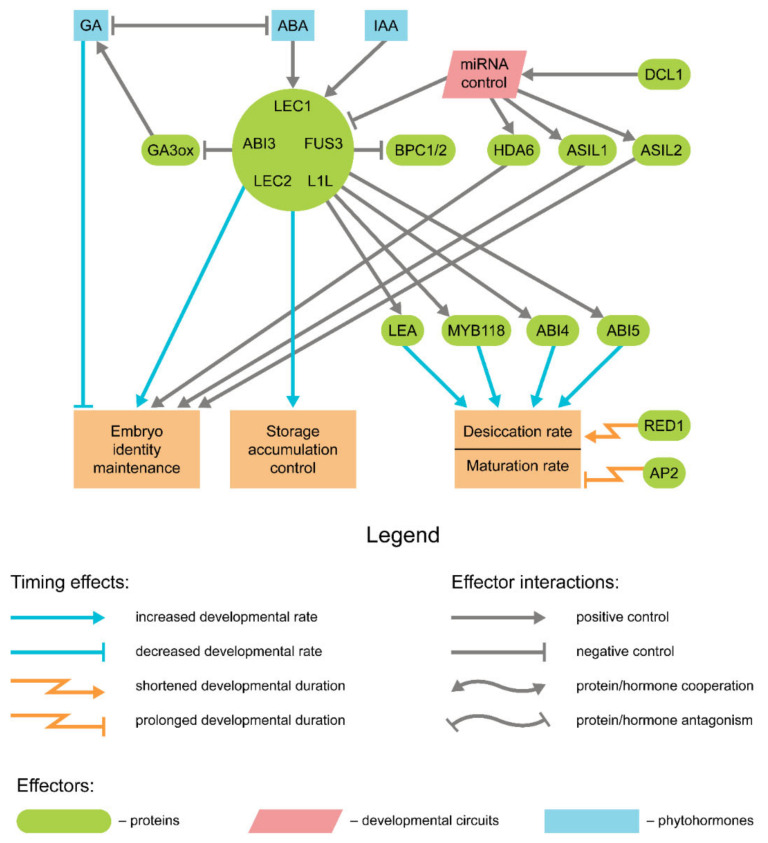
Key processes governed by LAFL factors in respect of maturation duration.

The governance over both the early (seed filling) and late (desiccation tolerance acquisition) maturation stage is shared by a set of transcriptional factors, namely, LEAFY COTYLEDON1 (LEC1), LEC1-LIKE (L1L), ABSCISIC ACID INSENSITIVE3 (ABI3), FUSCA3 (FUS3), and LEC2, together referred to as LAFL factors [19,21]. The latter three belong to a plant-specific B3 transcription factor family and thus are commonly denoted as ‘AFL-B3′, while LEC1 and L1L are attributed to the NF-YB family. Together these factors govern key processes accompanying the seed filling and desiccation (Figure 3). Despite a certain functional redundancy level [19], LAFL factors demonstrate distinct spatiotemporal patterns of occurrence and form complex regulatory loops themselves. The detailed account on the genetic control of seed maturation falls outside the scope of the present review and can be found elsewhere [17,18,19,20,21]. The principal point here is that precocious expression of any of the LAFL factors itself triggers the transition to maturation and thus affects seed developmental rates dramatically [18]. Loss-of-function mutations of LAFL, in turn, lead to a drastic shortening of maturation and premature vegetative growth [88,89,90].

Hormonal control of LAFL functioning is predominantly exerted by the auxin and ABA, serving as positive regulators of maturation and GA repressing the maturation program in favor of vegetative embryo growth. In *Arabidopsis*, the expression of FUS3 was found to also be positively regulated by auxin [91]. In turn, FUS3 positively regulates ABA synthesis and represses that of GA, thus securing the maturation onset [92,93]. ABA was shown to affect seed maturation as a part of the GA/ABA ratio, which reduces upon ABA concentration peaks [91]. The decrease of the GA/ABA ratio is further bolstered by repression of the active GA forms’ synthesis by LAFL factors. LEC2 and FUS3 were demonstrated to bind directly to the promoter elements of *AtGAox3*, negatively affecting its expression [94]. Somewhat counterintuitively, GA undergoes a short concentration peak during seed maturation as well, derepressing the LEC1 activity in the embryo and leading to further auxin accumulation [95]. To disentangle the complex functions of these hormones and delineate their functions in maturation control, auxin and ABA were proposed to operate through forming yet another concentration ratio [51].

The contribution of LAFL factors to seed maturation can be traced right to its onset at the transition phase, at which they promote the formation of the epidermis in *Arabidopsis* [96] and transfer cell layer in legumes [97]. Before that, LAFL gene expression is actively repressed at the transcriptional level by specific microRNAs (miRNAs) [24]. In this regard, the genes related to miRNA processing or maturation, such as *DICER-LIKE1* (*DCL1*), may affect the maturation timing. Weak *dcl1* mutants of *Arabidopsis* demonstrate precocious seed maturation due to the earlier activation of *L1L*, *LEC2*, and *FUS3* genes as well as their target genes, while the *LEC1* expression was downregulated [24,25]. The observed effects referred either to upregulation of *SQUAMOSA PROMOTER-BINDING PROTEIN-LIKE* (*SPL*) *10* and *11* transcription regulator genes [24] or to repression of genes encoding *ARABIDOPSIS 6B-INTERACTING PROTEIN1-LIKE (ASIL) 1* and *2* transcription factor and HDA/SIL histone deacetylase [25]. In the latter case, the *asil1* and *asil2* mutants, as well as all combinations of double mutants formed by these genes and SIL, demonstrate precocious maturation [25]. A similar effect is observed in double mutants for *E2FA* and *B* genes, although in this case, the onset of maturation overlaps with cell divisions [98]. It is also noteworthy that ectopic expression of *FUS3* may lead to similar developmental delay in embryo, as in the case of *Arabidopsis* mutants impaired in *BASIC PENTACYSTEINE* (*BPC*) *1* and *2* genes which produced FUS3 in the endosperm [99].

The downstream component of LAFL signaling may also affect the seed development rate. Ohto and colleagues [100] demonstrated that the duration of seed filling is longer in *ap2* mutants of *Arabidopsis* as compared to the wild type. *AP2* encodes a transcription regulator mostly associated with endosperm size control in seeds (see below); thus, prolonged seed filling duration may indicate both an indirect influence of endosperm growth on later embryo development or versatility of AP2 functions in *Arabidopsis* seeds. This prolongation is positively correlated with both the seed size and the cotyledon storage content [101,102]. Overexpression of another LAFL-governed gene, *AtMYB118*, leads to the premature desiccation of seeds residing at the distal fruit end [103]. Delayed desiccation and decreased seed tolerance to desiccation-associated stress were reported for *Arabidopsis* mutants affected in the *RED1* gene, although the functions of the respective protein are currently unknown [104]. Additionally, a certain impact was proposed for the LATE EMBRYOGENESIS ABUNDANT (LEA) proteins’ production. LEA proteins accumulate in desiccating seeds to ameliorate the impact of dehydration on cellular components [105]. Since active transcription of LEA genes significantly precedes the appearance of respective proteins in *M. truncatula* [106,107], it was proposed that the hiatus between LEA mRNA transcription and protein translation may fine-tune the onset of desiccation in response to environmental conditions. In *Arabidopsis*, the loss-of-function mutations in *EARLY METHIONINE-LABELLED* (*ATEM6*) gene encoding for a group 1 LEA protein result in a premature acquisition of desiccation tolerance alongside with a general acceleration of seed maturation, which can be mitigated by the overexpression of another group LEA gene, *ATEM1* [108,109].

Apparently, the desiccation stage is retained in most of the seed plants but is not mandatory. About 8% of flowering plants form seeds intolerant to drying [110], which sprout immediately upon dissemination or, in the most extreme case, inside the maternal fruits (e.g., being viviparous). Such seeds are commonly divided into two groups: recalcitrant seeds demonstrating intolerance towards drying to less than 90% relative humidity and intermediate seeds withstanding drying up to 30% relative humidity [111]. The terminally recalcitrant seeds bypass most desiccation hallmark processes, such as oxidative metabolism cessation [112]. However, accumulation of specific LEA proteins can be detected in maturing recalcitrant seeds of several species [113,114]. The functions of these proteins and their contribution to desiccation tolerance features in recalcitrant seeds remain elusive, although a limited tolerance towards faint drying has been reported [115]. Finally, some plants produce dimorphic seeds, in which seed morphs differ by the rate of desiccation tolerance and mucilage production [116]. Since most of the desiccation-associated programs are governed by ABA, alterations of ABA signaling are expected to cause either recalcitrance, viviparity, or both. This notion is supported by the evidence that intrinsically viviparous seeds are depleted in ABA levels during maturation [6,7] and that the external application of ABA partially arrests viviparity [117]. Moreover, in lemon (*Citrus limon*), which begets the intermediate seeds, GA synthesis inhibition by paclobutrazol resulted in the promotion of ABA synthesis and LEA protein accumulation followed by the establishment of desiccation tolerance [118], indicating that GA/ABA ratio rather than ABA concentration solely conditions seed desiccation. At the same time, mutations in genes encoding for ABA biosynthesis enzymes and ABA response factors have been reported to cause desiccation intolerance in orthodox seeds of both monocots and dicots [119,120,121]. A similar phenotype is observed in several LAFL mutants underpinning their importance for both early and late maturation progress [4,122].

## 5. Endosperm and Seed Coat Development

**Figure 4:**
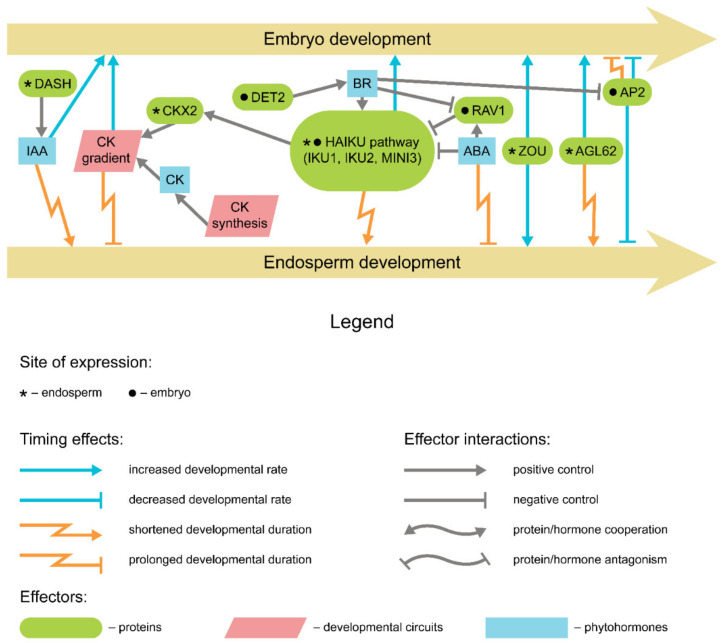
Key regulators of endosperm development timing and their effect on embryo development timing.

The molecular programs governing endosperm development may bear independence from those controlling embryo development, and vice versa. Such independence is apparently intrinsic for cruciferans, judging by the data obtained for *Arabidopsis* [60]. Despite this, endosperm may still retain its influence on embryo and overall seed developmental timing by setting physical constraints on seed size and cell number or through the impairment of nutrient transport (Figure 4). The impact of proper endosperm development on embryo growth and overall developmental timing is illustrated by *MINISEED3* (*MINI3*) and *IKU2* gene mutations belonging to the HAIKU signaling pathway [22,123]. These mutants demonstrate precocious endosperm cellularization, slowed embryo development, and a comparatively smaller embryo (and, by proxy, seed) size. Similar effects were observed for *AGL62* orthologs mutations [124,125]. *AGL62* product is also involved in both endosperm cellularization arrest and auxin export to seed coat [126], which may set an additional constraint on seed size and viability in the latter case. In *M. truncatula*, mutations of the *DASH* gene lead to disruption of auxin efflux from the pod at constitutive levels of maternal auxin synthesis, which leads to abnormalities in endosperm [127]. *dash* mutants bear smaller seeds, and their embryo development is either delayed or aborted depending on the mutant allele.

The early endosperm development has been shown to be positively regulated by cytokinin signaling [128]. Endogenous cytokinin levels emerge at the chalazal domain of endosperm [129,130], while at the micropylar pole expression genes encoding for cytokinin oxidases (CKXs) is promoted by HAIKU pathway components, leading to the cytokinin gradient established in endosperm along the chalazal-micropylar axis [123]. Counterintuitively, both the *ckx* mutants and cytokinin-insensitive mutants were found to beget large seeds with regular seed development timing in both monocots and dicots [123,131,132,133]. A plausible explanation for this discrepancy indicates that the global cytokinin signaling impairment alters the distribution of carbon supplies within the plant, increasing the nutrient sink directed to the generative tissues (reviewed in reference [134]). HAIKU pathway itself is at least partially controlled by brassinosteroids in both seed coat and filial tissues, with brassinosteroid-deficient *det2* mutants of *Arabidopsis* demonstrating both embryo retardation and reduced seed size [135]. ABA then represses the HAIKU pathway, thus suspending the endosperm development. ABA deficiency triggered by *aba2* mutations delays the endosperm cellularization resulting in prolonged seed development and increased seed size [54]. In addition, the ABA-related transcription regulator RAV1 was found to repress the HAIKU pathway in *Arabidopsis*, but the exact impact of null mutations on seed developmental timing was not assessed [136].

Most eudicots deposit storage compounds in cotyledon cells, which implies redundancy of a well-developed endosperm [137]. To this end, endosperm undergoes gradual absorption by the growing embryo during seed filling. *Arabidopsis* mutants of *RETARDED GROWTH OF EMBRYO1* (*RGE1*), also known as *ZHOUPI* (*ZOU*), exhibit developmental retardation starting after the heart stage and a decreased seed size due to the incomplete endosperm resorption [138,139].

The effects of endosperm on embryo development and, therefore, seed development timing partially resemble those exerted by the seed coat. The *ap2* mutants of *Arabidopsis* and rapeseed (*Brassica napus*), which have their seed filling stage prolonged (see above), also demonstrate the prolonged pre-storage resulting in longer seed development and increased seed size, and this effect is claimed to be similar to that of *arf2* mutation affecting seed coat proliferation [100,140]. In fact, the AP2 transcription factor negatively controls seed development by restricting cell proliferation in both seed coat and endosperm [100]. The similarity between ARF2 and AP2 functions is underpinned by their shared negative control by brassinosteroid signaling [135]. A similar effect was observed in *Arabidopsis* seeds ectopically expressing *FUS3* in endosperm tissues, although adverse effects lead to decreased seed viability in this case [99]. For the seed coat, the effect on embryo development timing was also demonstrated by obtaining *nars1* and *nars2* mutants of *Arabidopsis* [141]. The transcription factors encoded by these genes operate in the seed coat and are presumably involved in nutrient transport and programmed cell death in inner seed coat layers. Notably, the endosperm development and breakdown were also delayed in *nars* mutants, suggesting a partial concordance of embryo and endosperm development in this case.

## 6. Two-Membrane Organelle Functioning and Energy Metabolism

Plastids are involved in various cellular processes, of which photosynthetic activity poses one of the most crucial. The importance of proper plastidial maintenance for seed development is further prompted by the wide distribution of the so-called stay-green seeds capable of photosynthesis [142]. Depending on embryogenesis timing and seedling viability, mutants impaired by plastidial gene mutations were suggested to fall into four categories ranging from lethal embryo specimens to retarded at embryogenesis yet fully viable and fertile mutants [143]. The latter provides individual variations for seed development timing and comprises mutations affecting genes with partially redundant or dispensable functions. In *Arabidopsis*, these include weak *clpr1*, *clpr2*, *clpp4*, and *clpp6* mutations of chloroplast Clp protease family genes [143] and mutations in genes encoding ClpB3 plastidial chaperone [144], Tic40 inner membrane translocon subunit [145], FtsH protease [146]. Of nuclear genes involved in plastid functionality, those encoding the ATPC1 gamma subunit of plastidial ATP synthase [147] and IM terminal oxidase [148] were suggested to impact seed developmental timing [143]. It is possible that for the null mutations displaying embryo lethality, permissive weaker alleles also exist, as in the case of the *PPR2* gene of *Arabidopsis* [149], therefore providing more material for speculation on the plastidial impact on seed development timing 

Basically, the mitochondrial role in seed development is usually perceived in the light of embryonic energy status [150]. Judging by the scarce evidence resent, proper mitochondria functioning may influence seed development timing through other mechanisms. In *Arabidopsis*, the mutation in *ETHE1* locus encoding for mitochondrial sulfur deoxygenase results in prolonged seed development and smaller seed size due to redistribution of storage composition, ABA deficiency, and distorted embryo development [151]. ETHE1 is involved in the amino acid turnover in the absence of carbohydrates [152], which explains the observed developmental delay under light deprivation. *Arabidopsis ca1ca2* double mutants impaired in mitochondrial electron transport demonstrate severe embryogenesis delay and absolute seedling lethality upon germination [153]. The observed phenotype was proposed to be connected with both respiratory insufficiency and elevated levels of reactive oxygen species (ROS). The importance of ROS scavenging is underpinned by the similar phenotype of *Arabidopsis pex10* mutants impaired in peroxisome biogenesis [154] and a more permissive delayed embryogenesis phenotype in *str1 str2* mutants impaired in RBOH-associated mitochondrial proteins [155]. Provided that ROS can serve as signaling molecules [156,157], it is possible that ROS formation, scavenging, and signal transduction may represent the prominent players in novel embryonic timing control mechanisms as well.

## 7. Metabolic Control of Seed Development

**Figure 5:**
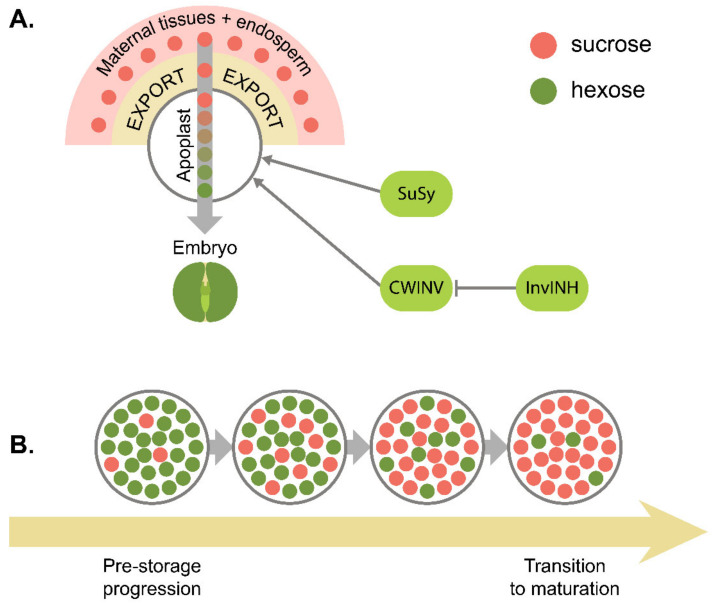
A representation of the ‘invertase control hypothesis’ as proposed for legumes. (**A**) General scheme of low-molecular sugars’ flow in legume seeds at the patterning phase. (**B**) Dynamics of hexose and sucrose sugars in embryonic tissues. A decrease of cell wall invertases and SuSy activity leads to a fall of hexose/sucrose ratio, which serves as a metabolic signal for the maturation onset.

Low-molecular carbohydrates, especially glucose and sucrose, exert a versatile function in seed development. Apart from being involved in energy metabolism, carbon supplement, and starch synthesis, glucose and sucrose were shown to serve as signal molecules. Basically, sugars are transported in the form of sucrose, which is first exported via SWEET family transporters and imported through SUF sucrose/H^+^ cotransporters [158]. Apart from that, endosperm can utilize apoplast-bound sucrose by importing it through SUC transporters in *Arabidopsis* [159]. Along this pathway, sucrose can be cleaved into glucose and fructose by invertase or into fructose and UDP-glucose by sucrose synthase (SuSy) [158]. At the initial stages of the seed development, sucrose is actively hydrolyzed, and the resulting high hexose/sucrose ratio serves as a developmental signal controlling the transition between stages. This model, referred to as the ‘invertase control hypothesis,’ or ‘sugar switch hypothesis’ [12,158], has been proven for legumes [27]. The hexose/sucrose ratio decrease in this model is consistent with maturation progression [12,28] (Figure 5). Once the transfer cell layer is established, the embryo switches to the uptake of sucrose as a primary carbon source and material for starch synthesis, as shown in *V. faba* [160]. These cells are marked with specific patterns of carbohydrate transporter-encoding gene expression, including the upregulation of *SUT1*, *AHA1*, and *SBP* sucrose transporter orthologs [161]. The formation of transfer cells, being itself a marker of transition to maturation, is also dependent on the carbohydrate status [28,161,162,163,164]. In *V. faba*, exposure to high hexose levels was demonstrated to initiate the transfer cell specification [165,166], while the excessive sucrose application prolonged callus-like embryo growth and postponed the transition [164,165]. In *M. truncatula*, a similar delay of transition was induced by increased auxin levels [84]. It should be noted, however, that independence of the transition onset from hexose/sucrose ratio was demonstrated for tobacco [167], *Brassica napus* [168], and *Arabidopsis* [60], undermining the applicability of invertase control hypothesis outside the Fabaceae family. In this regard, the data acquired for *Arabidopsis* mutants (see below) may be explained by reasons unrelated to developmental timing control per se.

The conformity to the invertase theory notwithstanding, both sugar transport and catabolism in the apoplastic space may exert their effect on seed developmental progress. The respective mutations affect sucrose transport through seed tissues and include, among others, a delayed seed development during embryogenesis and reduced seed weight. In *Arabidopsis*, only triple *sweet11;12;15* mutants exhibit pronounced developmental retardation at both embryo morphogenesis and maturation stages [169], while in *G. max*, severe embryogenesis retardation and high levels of seed abortion are achieved in single *gmsweet15-1* and *gmsweet15-2* mutants [170]. For *suc5* mutants of *Arabidopsis*, a similar yet much slighter effect was observed [159]. However, this exact SUC member was further demonstrated to be involved predominantly in biotin transport, and the observed retardation phenotype may be attributed to reduced triacylglycerol accumulation instead [171]. Consistent with the notion of hexose/sucrose ratio control, the prolonged expression of *InvINH1* encoding invertase inhibitor in *Arabidopsis* seeds brings about a transient retardation of embryo development at the pre-storage stage [172]. Conversely, during the seed maturation, the ectopic activity of acid invertases leads to a significant shortening of the filling stage in wild *Cicer judaicum* compared to domesticated chickpea (*Cicer aeretinum*) [173]. In SuSy-impaired mutants of species in question, no developmental delay has been reported so far, presumably due to the redundancy of individual SUS genes [174]. However, the earlier onset of SuSy activity was reported in thermotolerant, rapidly maturing accessions of greengram (*Vigna radiata*) [175]. In addition, the prolonged pre-storage phase in *ap2* mutants of *Arabidopsis* was shown to correlate with the elevated hexose/sucrose ratio [100].

Sugar signaling is tightly intertwined with hormonal regulation pathways, including those of auxin and ABA (reviewed in reference [176]). The sucrose sensing impairment invokes a phenotype similar to that of ABA-insensitive mutants [177]. The interplay between ABA and sugar signaling is maintained through two central control circuits. One is mediated by SUCROSE NON-FERMENTING-1-related kinase (SnRK1) (reviewed in reference [12]). SnRK1 acts as a catalytic subunit, often referred to as an α-subunit, of SNF1/AMPK complex coupling stress response and metabolic activity in various organisms [178,179,180]. In *P. sativum*, a decrease in SnRK1 expression leads to an extended pre-storage phase in a manner similar to that of ABA-deficient mutants, suggesting growth retardation [181]. Further inspection revealed that PsSnRK1 directly promotes embryonic ABA synthesis [182]. An even tighter link between SnRK1 and ABA signaling stems from the fact that SnRK1 directly activates FUS3 via phosphorylation in *Arabidopsis* [183]. Consequently, the mutations in genes encoding SnRK1 α-subunits and mutations impairing phosphorylation site in FUS3 lead to provoked a similar phenotype marked with the slowed embryogenesis progress, reduced maturation stage, and frequent seed abortion [183]. The other crucial sugar signaling circuit revolves around trehalose and its precursor, trehalose-6-phosphate (T6P). These molecules serve as both positive indicators of sucrose availability and negative regulators of its synthesis (see paper [176] and references therein). T6P synthesis from UDP-glucose and glucose-6-phosphate is catalyzed by trehalose 6-phosphate synthase (TPS), whose proper activity was demonstrated to be crucial for embryogenesis progress in *Arabidopsis*. *tps1* mutants are marked with slowed cell division rate and delayed embryo development at pre-storage, frequently followed by embryo abortion at the torpedo stage [29,184]. At the molecular level, this effect is pronounced through the decreased levels of sucrose, lipids, and storage proteins in seed tissues and the upregulation of ABA-responsive genes [29]. On the contrary, the TPS overexpression leads to sucrose and ABA insensitivity [185].

While legumes mostly deposit nutrients in the form of storage proteins, it was shown that impairment of starch formation affects protein content in *P. sativum* [186]. Moreover, in *Vicia narbonensis*, antisense inhibition of the gene encoding for ADP-glucose pyrophosphorylase (AGP) resulted in a prolonged seed filling compensating low starch depositions and leading to increased storage protein level [187]. The accumulated starch, in this case, might serve either as an energy supply for seed metabolism or a carbon source for protein synthesis. In oilseed rape (*Brassica napus*), whose seeds store carbon mostly in the form of triacylglycerols, a similar effect of AGP repression was documented regarding oil biosynthesis [188]. 

Compared to carbohydrates, the metabolic signaling of nitrogen storage in temporal control appears less clear. Basically, developing seeds rely on the maternal nitrogen supplies, with embryos left devoid of nitrogen influx growing incapable of attaining storage protein accumulation in *M. truncatula* [189]. Overexpression of the genes encoding phosphoenolpyruvate carboxylase (PEPC) in *V. narbonensis* (moor’s pea) apparently leads to a preferential allocation of carbon skeletons and nitrogen towards amino acid synthesis, which results in both elevated storage protein content and prolonged seed maturation [190,191]. Among the observed effects, an increase of *ABI3* expression was recorded, although the ABA levels were found to be elevated only at the pre-storage phase. In addition, several mutations affecting translation machinery have been reported to impact the seed development rate so far. Semi-dominant *rpl27a* mutation in *Arabidopsis* negatively affects the pace of embryo growth and patterning, presumably due to distorted auxin distribution [192]. The mutations in two other ribosomal protein genes, *RPL18aB* and *RPS5A* (*AML1*), of *Arabidopsis* trigger even more severe consequences, including complete developmental arrest during embryogenesis [193,194]. The observed effects of the latter mutations may be attributed to a general debilitation of cell viability and proliferation rate during embryogenesis rather than to specific effects of storage protein accumulation. Nevertheless, precocious lines of *P. sativum* are characterized by altered expression dynamics of genes encoding for seed storage proteins that might represent an important mechanism underlying developmental acceleration [195]. Finally, in the light of the recent discovery of amyloid aggregates formation by the pea seed storage protein vicilin [196] that seems to represent a conservative feature of the seed storage globulins not only in legumes but rather across land plants [197], it is also likely translation rates together with protein aggregate assembly/disassembly dynamics may affect the progression of seed development.

Similar to that of protein biosynthesis, the effect of oil and lipid synthesis on the seed developmental rate remains elusive. In *Arabidopsis*, which deposits oils as primary storage compounds, mutations in genes encoding pyruvate kinases (*PKP1/2*) and master regulator of fatty acid synthesis WRINKLED1 (*WRI1*) displayed embryo growth retardation pronounced at the pre-storage stage as well as slightly reduced seed size [30,31]. Given the aforementioned effect of AGP repression on oil production [188], oil synthesis might also have an indirect effect on seed development via interference in carbon partitioning. As for the lipids unrelated to the oil storage, the weak mutation in the *PECT* gene resulted in the delayed embryo growth and development due to phosphatidylethanolamine synthesis impairment in *Arabidopsis* [198]. All in all, the role of oil and protein synthesis in the metabolic control of seed development requires further investigation.

## 8. Environmental Factors Affecting Seed Development Rate

External stimuli affect both maternal and filial mechanisms conditioning the seed development. Among these stimuli, the major role is usually attributed to abiotic factors such as temperature, humidity, luminosity, and supplies of available nutrients, while biotic factors, such as interactions between plants and microorganisms, are less studied. 

Most of the studies suggest that favorable conditions lead to longer seed development and larger seed size, while in a stressing environment, seeds tend to have a shorter developmental cycle [199,200] due to alterations in carbon and nitrogen flux partitioning. Assuming that the seed size is mostly determined by the number of cotyledon cells (see the ‘Cell Proliferation During Embryogenesis’ section), cell division rate is expected to be positively correlated with the activity of nutrient sink to developing seeds [48,201]. In this regard, the intrinsic constraints for seed growth comprise cell number, mean cell size, and storage capacity [201]. For *P. sativum* and *G. max*, the primary causes of reduced seed size were proposed to be restricted cell proliferation and expansion [48]; however, the data regarding environmental effects on the pre-storage stage progress seem to be inconsistent [202,203,204]. In legumes, the duration of seed development is reduced in response to insufficient nutrient sinks resulting in smaller seeds. In several legume crops, including *P. sativum*, *G. max*, *Lupinus albus* (white lupin) [205], *Vigna unguiculata* (cowpea) [206], and *C. aeretinum* [207], the exposure to supraoptimal temperature reduced the time of seed maturation resulting in smaller seed size and lower weight. In *G. max*, the increased temperature negatively affected cell division rate indicating both a prolonged pre-storage phase and reduced cotyledon cell number [204]. In lentil (*Lens culinaris*), heat and drought stresses coupled together led to a decrease in seed filling rate and duration; however, the concomitant decrease in seed size was attributed to a reduced storage content [208,209]. The increased rates of seed filling at higher temperatures were demonstrated to be related to nitrogen uptake and remobilization in *P. sativum* [34]. In *V. radiata*, both higher ambient temperature and reduced photoperiod were found to accelerate seed maturation at the cost of seed size and nutrient composition in thermosusceptible accessions [175]. This effect was not observed in thermotolerant accessions with stable high seed yields, presumably due to early sucrose synthase activation and enhanced production of Hsp101 molecular chaperones [175]. A similar phenomenon was observed in perennial babysbreath (*Gypsophila paniculata*, family Caryophyllaceae), whose seed maturation phenology was accelerated by elevated ambient temperatures [210]. Apart from the direct influence of heat or cold stress, ambient temperature affects seed development through modulating atmosphere carbon availability [32,33,201,211], with elevated temperatures causing a shortage of carbohydrate supply.

Apart from abiotic factors affecting seed maturation timing, surrounding organisms might influence the process of maturation. Dicots can establish complex symbioses with soil microorganisms, including arbuscular mycorrhizal fungi [212,213], plant growth-promoting bacteria [214], and, in the case of certain dicot families, nitrogen-fixing bacteria of the Rhizobiales order [215]. Although the mechanisms underlying their function and specificity have certain similarities, they play different roles. Mycorrhizal fungi are mostly responsible for the nutrient uptake from soil [216,217], nodule bacteria fix nitrogen from the atmosphere [218,219], and growth-promoting bacteria perform microelement uptake, produce growth hormone, and promote resistance to pathogens [220].

In *P. sativum*, the uplifted rates of maturation-associated protein production may be accompanied by pronounced temporal changes upon the establishment of symbioses. Mamontova and colleagues [221] demonstrated that the highly effective interactions with mycorrhizal fungus *Rhizophagus irregularis* and root nodule bacterium *Rhizobium leguminosarum* positively affected the accumulation of storage and desiccation-associated proteins upon combined inoculation. The observed differences were suggested to result from the prolongation of the seed filling stage in the inoculated plants. It is hard to determine whether the effect was brought about by a particular symbiont. Further studies revealed that establishing mycorrhizal symbiosis was likely to prolong the seed filling stage resulting in a longer seed filling and higher yield [222]. The exact mechanisms behind the effect of mycorrhiza formation, however, remain poorly understood. The positive relationships between phosphorus uptake and seed dry mass have been shown in *G. max* [223], suggesting that the increased phosphorus influx may prolong seed filling. Speaking of nitrogen-fixing bacteria, the association with Rhizobia was demonstrated to promote seed biomass [224] and viability [225,226] in several legume species, albeit the effect was not retained under drought and temperature stresses [34,227]. 

The data on the effects of seedborne symbionts and parasites on seed development are scarce since the impact of seed-associated microorganisms is usually studied with respect to seed post-dispersal viability and further seedling development [228]. However, there is certain evidence that seedborne symbionts and parasites affect pre-germination seed development timing as well. The seeds of *M. truncatula* infected with *Xanthomonas alfalfae* and *Xanthomonas campestris* were found to lag in development starting from either seed filling or desiccation stages, with most of the ABI3 targets being significantly downregulated [36]. Conversely, susceptibility to *Xanthomonas axonopodis* was reported to cause late seed maturity and lower seed yield in *P. vulgaris* [229]. Similarly to mutualist symbionts, further investigations of molecular mechanisms of seed infections might provide new evidence of pathogen influence on the duration of seed development.

## 9. Is There an Integrative Scheme of Seed Development Timing Control?

**Figure 6:**
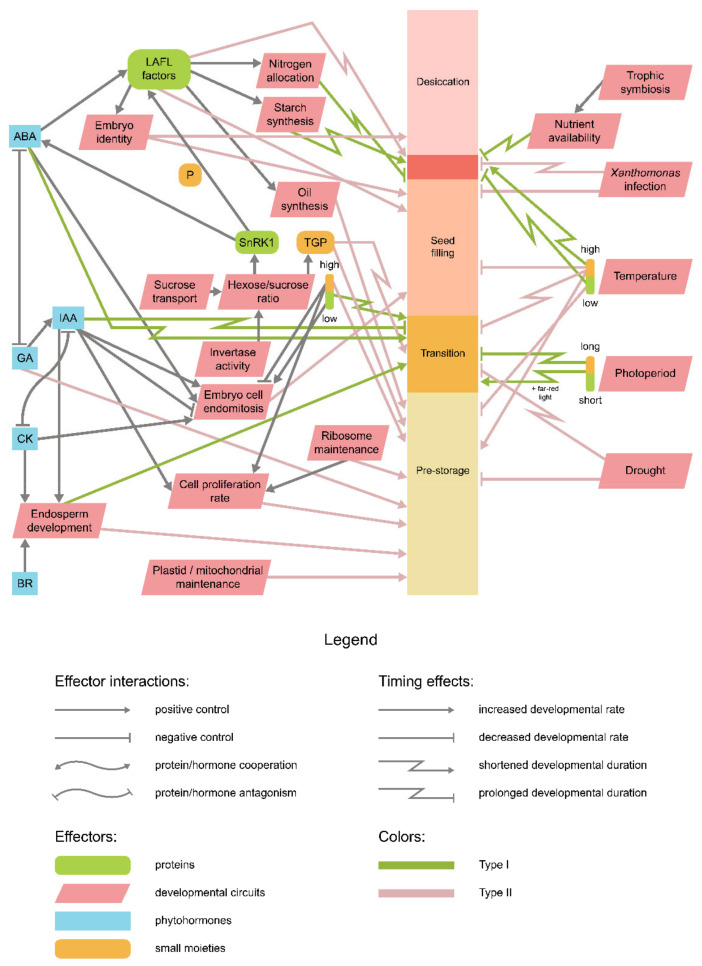
The integral scheme highlighting the principal components of dicot seed development timing control. See legend for arrow color/shape meaning.

Having analyzed the data gathered, we first pried whether the discussed examples could be divided by any distinctive features (as reflected in Figure 6). Following the notion that seed size and developmental timing are claimed to correlate positively in at least some plant species [48,49,230], we used this criterion to delineate the examples into two groups. The first group comprises examples pertaining to positive correlation, while the other consists of examples in which the correlation was either negative or absent. Hereafter we will refer to the mechanisms shared by the assorted examples as type I and type II developmental timing control alterations, respectively. 

A positive connection between seed size and duration of development results mostly from a stage succession delay rather than developmental deceleration. Apart from these loci, several loci are known to regulate the seed size in a similar manner, including *DA2* and *BIG BROTHER* genes encoding ubiquitin ligases [78,231], and *KLUH/CUP78A5* cytochrome P450 oxidase gene [232] in *Arabidopsis*. A wider list of similar genes for both monocots and dicots can be found in a recent review by Li and colleagues [233], with particular mechanisms observed in legumes further elucidated in a paper by Ochatt and Abirached-Darmency [81]; however, there are no data revealing the influence of most loci on the duration of seed development in respective species.

Notably, the developmental alterations that belong to type I are manifested in wild type plants under different environmental conditions, including varying illumination levels [35,175], temperature [34,175], and nutrient availability. A ‘tradeoff’ between phenological traits, including the time of flowering and seed production properties such as average seed size, seed number, and seed filling rates, has been proposed to be an adaptive strategy allowing plants to fine-tune the allocation of resources between vegetative and reproductive development. In the aphoristic triangular-shaped model proposed by Primack [234], the seed size is considered as a function of seed filling (or related phenological traits) with a multiplier that has an upper limit. Closer to the original wording, the longer seed filling period does not necessarily result in larger seeds, but its deceleration inevitably reduces the seed size. Recently, Segrestin and colleagues analyzed the relationships between seed dimensional properties and phenological traits in 139 species from the Mediterranean region and found that this dependence remains linear in annual species except for perennial and woody forms [235].

Direct interaction of seed size and development time may provide certain ecological flexibility. Short generation time, larger seed quantities, and smaller seed size are commonly associated with r-strategy in plants that undergo stress or adapt to new habitats [236,237,238], while K-strategy involves the production of a smaller number of large seeds undergoing prolonged development [239,240]. In a broader sense, reproductive cycle compression is frequently reported for invasive species [237,241], although most works concentrate on the duration of the vegetative cycle. As variations in seed development timing within species may reflect the adaptation to contrast habitats, respective genotype-dependent differences are likely to provide resources for species evolution and divergence.

While the data on loci attributed to type I control circuits keep up well with their evolutionary and ecological rationale, the majority of mutations discussed in this review appear to be strikingly inconsistent with the proposed size-duration relation model. Certain mutations negatively affect seed viability by disrupting phytohormonal signaling [72,73,135], cell cycle progression [59,61,64], or metabolic supplies [30,31,172]. Most of these mutations manifest themselves at the pre-storage phase, in agreement with the data indicating a crucial role of embryo cell number and volume in determining the final seed size [48,242]. Being affected by the distortion of this type, seeds progress through the development with a smaller number of embryo cells with a subsequent decrease of seed viability. Due to their detrimental nature, type II alterations are unlikely to pose any source of adaptive mechanisms for their hosts. Moreover, at some point, any mutation reducing cell proliferation without negative effects on embryo viability could be involved in a type II control circuit. In notation acquired adopted in *Arabidopsis* developmental biology, those type II mutations that affect early seed development might be considered as permissive EMBRYO-DEFECTIVE (EMB) genes’ mutations [243]. One of the possible explanations for retardations in type II mutants is decoupling of the absolute (‘chronological’) and relative (‘developmental’) time aspects of embryo development. In animal developmental biology, the nature of phenotypes resulting from this decoupling remains one of the least understood matters of developmental timing [244]. In plants, this concept remains mostly obscure, although the data from *Arabidopsis* indicate that in this plant relative and absolute timelines of seed development are uncoupled with developmental time defining the developmental state [60,100].

To fully understand the evolutionary impact of both types of timing alteration, one may also apply to the concept of heterochrony (reviewed in references [245,246,247]). In a broader sense, heterochrony stands for alteration of either development duration, rate, or both, in a specimen compared to its ancestors. Depending on whether the duration of the ancestral developmental sequence is prolonged or shortened, the heterochronic events are referred to as peramorphosis or pedomorphosis, respectively. Each group is further divided into subcategories depending on the nature of the observed changes. Compared to animal developmental biology, in plant biology, the concept of heterochrony is claimed to be largely dismissed. Certain efforts, however, were made to inculcate the concept relating to plant development and evolution, leading to suggesting the leading role of heterochrony in land plant diversification [248] and flower shape evolution [249]. Similarly, the molecular bases for heterochronic events in plants have been studied in regard to a limited number of developmental events, such as juvenile-to-adult and vegetative-to-flowering transitions [246]. The respective pathway relies on two regulatory microRNAs, miR156 and miR172, which form a successive and antagonistic controlling circuit [246,250]. Compared to that, timing control of seed development remains hardly tackled by plant developmentalists. Few examples include the recent works on temporal coordination of embryo and endosperm development in *Arabidopsis* carried out by O’Neil and colleagues [60] and mutants impaired in seed maturation program [245,251,252].

Apparently, the heterochrony nomenclature provides suitable terms for the description of timing alteration types. For instance, type I refers to the progenesis-hypermorphosis heterochrony type, in which the overall developmental duration is prolonged (hypermorphosis) or shortened (progenesis), respectively, while developmental rate remains intact. Similarly, type II may be considered as a representation of neoteny-acceleration heterochrony. Unlike the previous type, this pair describes temporal changes instigated by the changes of developmental rate regardless of the ‘chronological’ duration of development. The cases of neoteny and acceleration in plants are mostly studied within the context of floral development [253], with one notable exception of endophyte development in the Rafflesiaceae family members [9]. However, temporal traits like time in days to flowering or to seed maturity have been reported to be negatively correlated with seed weight in *G. max* [38] and *P. vulgaris* [40]. Alternatively, adjusting the developmental speed might represent a mechanism underlying a non-linear dependency between seed size and phenology in perennial crops [235]. Further assessment of type II control circuits in natural variations of seed development timing and their potential benefits has several limitations: a) the works on embryogenesis retardation seldom account for overall seed development duration of mutant specimens; b) contrast phenotypes corresponding to strong or null mutations are usually used to demonstrate the role of the studied genes; and c) the effects of gain-of-function mutations or gene overexpression are reported less frequently compared to loss-of-function mutations.

In some of the reviewed cases, the apparent heterochrony might be rather considered as homeosis or an ectopic organ development [245]. This phenomenon arises, for instance, in the mutants with both distorted developmental timing and altered embryo patterning [254,255]. The difference between these homeotic mutants and the representatives of the aforementioned two types suggests a somewhat independent nature of the mechanisms leading to heterochrony in these cases. The observed cases of viviparity in orthodox seeds [119,120,121,122] or, vice versa, developed desiccation tolerance in recalcitrant or intermediate seeds [117,118] imply that the loss of embryo identity after seed filling and prevention of late maturation may lead to viable seed phenotypes and novel evolutionary strategies.

To sum up, the previously proposed criterion of size-to-duration correlation in dicot seeds leads to the dissection of three distinct trends of developmental timing regulation. Indeed, these types do not reveal the common molecular basis for the grouped events. In this regard, the proposed division cannot be perceived as a bona fide classification and does not point out the common molecular basis of these heterochronic events. However, certain commonalities within each type may be found: for instance, type I genetic mutations demonstrate similarity to adaptive responses to varying environmental stimuli, whilst type II examples affect seed development mostly during the pre-storage phase. Given the variety of mechanisms underlying these processes, as summarized in Figure 6, future studies may concentrate on the interconnections of mechanisms affected by respective mutations and those defining duration and rate of seed developmental stages.

## 10. Concluding Remarks

Judging by the data gathered, a wide variety of the affected regulatory pathways indicates that developmental timing control in seeds is mediated by complex mechanisms that, by now, cannot be simply reduced to a well-defined ‘heterochronic pathway.’ The diversity of the mechanisms resolving in temporal alterations urges the idea that such a pathway, if it ever exists, should be versatile enough to orchestrate numerous circuits of cellular metabolism. Similar to the heterochronic pathway controlling stage transitions during the vegetative cycle [250], small RNA-mediated mechanisms pose promising candidates for a putative seed timing control pathway. Following this suggestion, at least two further ramifications for future studies emerge. First, analogs of the heterochronic pathway controlling meristem initiation found in monocots suggest that the comparative approach could be exploited to elucidate the actual mechanisms underlying seed temporal plasticity. Because of the definitive differences between monocots and dicots regarding both their seed development [256,257] and the peculiarities of their vegetative heterochronic pathways [258], we deliberately restrained ourselves from drawing any examples from monocot species in this review; however, several reports indicate that processes denoted here as type I temporal alterations may arise in cereals to adjust their seed developmental timing to heat [259,260,261,262,263,264,265,266], moderate cold [267], drought [268], and mycorrhiza formation [269]. Second, these mechanisms may control a multitude of processes and act through numerous mediating components, which raises the necessity for large-scale gene expression studies involving the techniques of modern transcriptomics and proteomics. This might be especially essential for dissecting type I mechanisms as, in this case, developmental alterations are coupled with the environmental responses, which also involve a coordinated expression of numerous genes. It is also likely that control over seed heterochrony may be exerted by a transcriptional master regulator or a set of such regulators like those of the LAFL family; in this regard, mutations in the respective genes may also lead to homeotic alterations in embryo organ identity, allowing developing embryos to completely bypass several developmental stages. Such mechanisms may also be interconnected as microRNA and LAFL control circuits have been found to be interrelated in seed development [24,25]. Finally, the existence of distinct types of temporal plasticity, designated here as type I and type II, respectively, further prompts whether even two or more coupled regulators could exist simultaneously or whether the regulating pathway exerts control on both phase progress and succession. Whichever hypothesis future studies would adopt, we believe that these suggestions will provide a useful framework by which different types, causes, and outcomes can be delineated and evaluated.

## Supplementary Materials

The following are available online at https://www.mdpi.com/article/10.3390/ijms22179252/s1, Table S1: The list of genetic loci affecting seed developmental timing mentioned in this paper.
